# *Schistosoma haematobium* infection morbidity, praziquantel effectiveness and reinfection rate among children and young adults in Gabon

**DOI:** 10.1186/s13071-019-3836-6

**Published:** 2019-12-10

**Authors:** Jean Claude Dejon-Agobé, Jean Ronald Edoa, Yabo Josiane Honkpehedji, Jeannot Fréjus Zinsou, Bayodé Roméo Adégbitè, Mirabeau Mbong Ngwese, Ance Mangaboula, Bertrand Lell, Martin Peter Grobusch, Benjamin Mordmüller, Ayôla Akim Adegnika

**Affiliations:** 1 Centre de Recherches Médicales de Lambaréné, Lambaréné, Gabon; 2 Center of Tropical Medicine and Travel Medicine, Department of Infectious Diseases, Division of Internal Medicine, Amsterdam University Medical Centers, Location AMC, University of Amsterdam, Amsterdam, The Netherlands; 3 Department of Parasitology, Leiden University Medical Center, Leiden, The Netherlands; 4 Division of Infectious Diseases and Tropical Medicine, Department of Medicine 1, Medical University of Vienna, Vienna, Austria; 5 Institut für Tropenmedizin, Eberhard Karls Universität Tübingen, Partner Site, Tübingen, Germany; 6 German Center for Infection Research, Tübingen, Germany

**Keywords:** *Schistosoma* spp., Morbidity, Praziquantel, Egg rate reduction, Cure rate, Efficacy, Effectiveness, Reinfection, Incidence, Prevalence

## Abstract

**Background:**

Sub-Saharan Africa carries most of the global burden of schistosomiasis. To optimize disease control and reduce morbidity, precise data are needed for control measures adapted to the local epidemiological situation. The objective of this study is to provide baseline information on schistosomiasis dynamics, including praziquantel (PZQ) treatment outcome in children and young adults living in the vicinity of Lambaréné, Gabon.

**Methods:**

Eligible volunteers were included into a prospective longitudinal study. Urine filtration technique was used to detect eggs in urine for schistosomiasis diagnosis. Subjects were treated with 60 mg of PZQ once per month for three consecutive months, and the outcome was assessed by cure rate (CR) and egg reduction rate (ERR).

**Results:**

A total of 328 volunteers were enrolled in the study with a mean (± SD) age of 12.2 ± 4.7 years-old. The female-to-male ratio was 0.99. Out of 258 participants in total, 45% had schistosomiasis during the survey and 43% presented with heavy infections. The incidences of haematuria and schistosomiasis were 0.11 and 0.17 person-years, respectively. After the first and third dose of PZQ, overall ERR of 93% and 95% were found, respectively; while the CR were 78% and 88%, respectively. Both ERR (100 *vs* 88%) and CR (90 *vs* 68%) were higher among females than males after the first dose. The CR increased for both groups after the third dose to 95% and 80%, respectively. After the first PZQ dose, ERR was higher for heavy compared to light infections (94 *vs* 89%), while the CR was higher for light than for heavy infections (87 *vs* 59%). After the third PZQ dose, ERR increased only for light infections to 99%, while CR increased to 98% and 75% for light and for heavy infections, respectively. The reinfection rate assessed at a mean of 44.6 weeks post-treatment was 25%.

**Conclusions:**

The prevalence of schistosomiasis is moderate in communities living in the vicinity of Lambaréné, where a subpopulation with a high risk of reinfection bears most of the burden of the disease. To improve schistosomiasis control in this scenario, we suggest education of these high-risk groups to seek themselves a one-year PZQ treatment.

*Trial registration* clinicaltrials.gov Identifier NCT 02769103. Registered 11 May 2016, retrospectively registered. https://clinicaltrials.gov/ct2/show/NCT02769013

## Background

Schistosomiasis is considered the second most important parasitic disease after malaria [1]. It is a neglected tropical disease occurring frequently in sub-Saharan Africa where 85% of the worldwide infected population lives [2]. The disease is poverty-associated, particularly in rural areas where parasite exposure through contact with infested freshwater is frequent. Indeed, parts of the population pursue daily activities such as household chores, bathing, and fishing in potentially infested water. In such areas where reinfection is common [3, 4], the WHO recommends implementation of targeted treatment through large-scale treatment to reduce the burden of disease [5], and to prevent morbidity in later life [6]. Administration of treatment at least once a year reduces early (visible haematuria, anemia) and late (portal hypertension, hepatic fibrosis, bladder cancer) schistosomiasis-associated morbidity [5, 7].

Few drugs are available for treatment of schistosomiasis. Metrifonate is an antischistosomal drug indicated for the treatment of schistosomiasis and effective only against *Schistosoma haematobium* [8]; however, the drug is no longer commercially available [9]. Oxamniquine is another antischistosomal drug effective only against *Schistosma mansoni* [8] but due to its higher price, it is used as an alternative drug when PZQ treatment fails [10]. Praziquantel (PZQ) is currently the WHO-recommended drug of choice, effective against adult worms of all *Schistosoma* species [8] and is used for large-scale treatment. The antimalarial drug artemether also has antischistosomal activity, particularly on juvenile schistosome stages [11] and therefore could play a role in disease prevention as demonstrated by Utzinger et al. [12]. In combination with PZQ, artemether can be used to target all parasite stages during schistosomiasis treatment. Indeed, in comparison to PZQ alone, artemether-PZQ combinations have shown to reduce the prevalence of schistosomiasis in Egyptian children by half, and to reduce disease incidence [13]. However, in Lambaréné and surroundings that are endemic for both malaria and schistosomiasis, the first results were contradictory [14]. Moreover, the regular use of artemisinin, which is the most important antimalarial drug, might contribute to the development of malaria parasite resistance [11] and then jeopardise current malaria control and treatment efforts. Another antimalarial drug, mefloquine, is found to be active on all parasite stages [15] and able to consistently reduce egg excretion [16].

For treatment of *Schistosoma intercalatum*, *S. haematobium* and *S. mansoni*, the main species prevalent in sub-Saharan Africa [5], the recommended dose of PZQ is 40 mg/kg in one or in a split dose, administered 4 hours apart [17]. Due to the confection of the drug (600 mg tablets) and to the usual difficulty to assess patient weight accurately particularly for children during MDA campaigns, PZQ is rarely administered in the most appropriate dosage. Dose scales for praziquantel administration have been developed by the WHO to minimise under-dosage of the drug [7] and to ensure administration of doses between 30 and 60 mg/kg, which is within the dose range that is considered both safe and effective [7, 18].

The WHO recommended diagnostic gold standard for schistosomiasis are urine filtration and Kato-Katz techniques for urogenital and intestinal schistosomiasis, respectively. The objective is to confirm the diagnosis by detecting *Schistosoma* eggs in fresh urine or stool samples [5]. The continuing presence or absence of *Schistosoma* eggs in urine or stool samples is used to assess PZQ efficacy for schistosomiasis treatment. As such, cure rate (CR) and egg reduction rate (ERR) are the two endpoints commonly used and recommended to evaluate anthelminthic drug efficacy [19]. WHO defines the efficacy of anthelminthic drugs as “the effect of the drug against helminths, in isolation and under ideal conditions” [7]. However, the outcome of these two tests “may vary widely, even in efficacy trials in which the same drug is given at the same dosage under optimal conditions” [7]. Therefore, to enable comparison between studies, the WHO suggested guidelines when assessing anthelmintic drug efficacy [19]. With regards to schistosomiasis, some of these remain difficult to assess, notably the variability in egg output and excretion or preponderance of immature worms less susceptible to PZQ. We therefore think that PZQ efficacy can only be properly estimated from a large number of individual studies across a range of epidemiologically distinct settings.

Lambaréné, a semi-urban town in Gabon, and its surroundings are known to be endemic for schistosomiasis, with *S. haematobium* reported as predominant [20–22] and *S. intercalatum* reported occasionally. An overall prevalence of 30% was recently reported for Lambaréné surroundings [23], rendering the community as having a moderate schistosomiasis prevalence. Whilst the epidemiological picture becomes clearer, there is a lack of information on several epidemiological indicators of schistosomiasis and the impact of PZQ. Therefore, the objective of this analysis is to provide basic information in regard to the parasitological indicators of schistosomiasis in our study population. These indicators include prevalence and incidence of the disease. In addition, the impact of PZQ treatment and reinfection were assessed. This information is relevant for improving schistosomiasis control in the area.

## Methods

### Study site

**Fig. 1:**
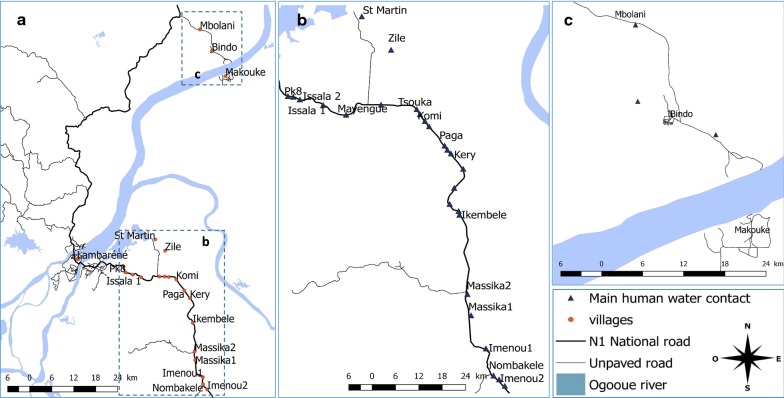
Map of Lambaréné, Gabon, and surrounding localities. **a** The main human-water contact points in the different study areas. **b** Zilé-PK villages. **c** Bindo village

The study was conducted at CERMEL, Centre de Recherches Médicales de Lambaréné, located in Lambaréné, Gabon. Volunteers were recruited from Zilé-PK area and Bindo village, two localities in the vicinity of Lambaréné where schistosomiasis is endemic. Zilé-PK villages is a set of villages located over 20 km (from PK 14 to PK 33) along the national road south of Lambaréné where many human-freshwater body contact points considered as schistosomiasis foci exist (Fig. 1), leading to a considerable level of urogenital schistosomiasis prevalence. Indeed, around 43% prevalence of schistosomiasis was reported, particularly in children [22, 23]. On the contrary, Bindo village, a remote locality located about 50 km north of Lambaréné presents very few human-freshwater body contact points which sustains around 15% schistosomiasis prevalence reported [22, 23].

### Study population

Volunteers aged 6–30 years-old, living in the study area for at least one year and without macroscopic haematuria and no apparent chronic disease during the screening phase were invited to participate in the study. School-age children and young adults are most afflicted by schistosomiasis, and the information from this population reflects best the community’s disease burden with the highest incidence. In the present study area the most common activities that bring young people into contact with open freshwater are fishing and household domestic activities, including water access for daily use.

### Study design

The present analysis is a sub-analysis of a longitudinal and prospective study designed to assess the effect of pre- and post-treatment of schistosomiasis with PZQ on malaria transmission. The study was conducted from June 2016 to November 2018. Following the screening phase, eligible participants were followed-up for 6 months. At month 6, schistosomiasis status was assessed for the whole study cohort according to the study procedure. Two study groups were therefore considered; study sub-group A, which included participants found to be positive, and study sub-group B, which included all participants found to be negative. Participants of sub-group A were treated during the 3 month treatment phase. From month 9 of the study (end of the study treatment phase), participants were followed for another 6 months (end of the study period), yielding a total follow-up time of 15 months. At the end of the study period, a second schistosomiasis status assessment was performed. In addition to the scheduled visits for schistosomiasis status assessment, participants were invited to actively visit the research centre in case of macroscopic haematuria or other health problems. In case of diagnosis of schistosomiasis and irrespective of the study phase, the participants had to receive a regimen of PZQ of 60 mg/kg body weight once a month during three consecutive months, administered under supervision of the clinical team. To minimize occurrence of adverse events related to PZQ treatment, participants were asked to eat before taking the drug. To assess treatment success, urine samples were collected four weeks after the first and third PZQ administrations.

### Sample size estimation

To address our main objective, the overall sample size to consider was simulated using the sample size calculation formula for cross-sectional studies [24]. Given that an overall prevalence of schistosomiasis of 30% has recently been reported for both study sites [23], and considering 1.96 standard normal variate and 5% precision, we estimated a minimum of 323 volunteers for inclusion in this survey. In addition, the minimum sample size recommended for PZQ efficacy assessment is 50 infected volunteers [19]. A sub-population was therefore analyzed for this secondary purpose.

### Laboratory procedures

The urine filtration technique as recommended by the WHO [25] was used to detect the presence of *Schistosoma* eggs in fresh urine samples. On days of sample collection, urine was collected between 10:00 and 15:00 h. For egg detection, the technique consisted of passing 10 ml of fresh urine through a micro-filter membrane of 10–12 µm (MF, Whatman, New Jersey, USA) using a syringe. The membrane was then transferred onto a glass slide, mounted on a microscope and read using a low-power objective (10×) of a light microscope. Reading of slides was performed by two independent experienced readers. The final result was reported as the number of eggs per 10 ml of urine after calculating the mean egg count obtained from the pooled results of both readers. In case of a quantitative (difference ≥ 20%) or a qualitative discrepancy between both readers, a third independent reader was required, and the mean of the two closest results was considered as the final result. For the diagnosis of urogenital schistosomiasis, urine samples were collected and processed over 3 consecutive days, unless the participant was found positive with at least 1 parasite egg in any sample before the second, or the third day of sampling. The participant was considered as negative if all 3 urine samples were negative for *Schistosoma* eggs. In addition, Rapid Dipstick (Combur test, Roche, Rotkreuz, Switzeland) was performed on each urine sample to detect evidence of haematuria.

### Statistical analysis

Data were managed using REDCap electronic data capture tool hosted at CERMEL [26]. The final database (Additional file 1) was exported into R version 3.4.4 for statistical analysis. Quantitative variables were summarized as the mean and standard deviation (SD) while qualitative variables were summarized as the proportion and 95% confidence interval (95% CI). Student’s t-test was used to compare continuous variables and Chi-square test or Fisher’s exact test was used to compare proportions. Significance of the *P*-values was set at < 0.05. With regard to the definition of the variables, a successful cure was defined as the conversion from positive to negative detection of *Schistosoma* eggs in the urine of treated individuals. Reinfection was considered as a new positive case, indicated by the presence of *Schistosoma* eggs in the urine of the participant who had previously been declared cured. In sub-group A, the CR was calculated as the percentage of volunteers cured among those treated, and the ERR was calculated on the basis of the total arithmetic mean egg counts after *vs* before treatment and expressed as a percentage as described elsewhere [19]. The intensity of infection was quantified as either light- or heavy-intensity infection using a threshold of 50 eggs per 10 ml of urine. In addition, all cases with visible haematuria were considered as heavy infections [7]. Person-time incidence rates were calculated using the total follow-up period of each participant and expressed in person-years. Incidence of visible haematuria was estimated among the whole cohort during the first study follow-up phase, while incidence of schistosomiasis cases was estimated in sub-group B during the second study follow-up phase.

## Results

### Study population

We included 351 volunteers in this study. Among them, 328 agreed to join the follow-up phase. The mean (± SD) age was 12.2 ± 4.7 years-old, with 75% of the participants being less than 15 years-old; the female:male ratio was 0.99. From the included volunteers, 79% were from Zilé-PK area (Table 1). Among the participants who joined the follow-up phase, 258 and 188 completed the first and the second study phase, respectively.

**Table 1:** Study population baseline socio-demographic characteristics and distribution of schistosomiasis cases. The proportion of schistosomiasis cases is distributed at the end of phase 1 and at the end of phase 2

	Study population characteristics at baseline			Schistosomiasis cases				*P-*value^a^
				End of phase 1		End of phase 2		
	*N*	%	95% CI (%)	*n*/*N*	%	*n*/*N*	%	
Overall	328	–	–	103/258	39.9	33/188	17.5	< 0.0001
Age								
6–8	69	21.0	16.7–25.8	16/61	26.2	10/44	22.7	0.86
9–11	102	31.1	26.1–36.4	34/80	42.5	13/66	19.7	0.006
12–14	81	24.7	19.8–29.4	32/69	46.4	7/50	14.0	0.0004
15–30	76	23.2	18.7–28.1	21/48	43.8	3/28	10.7	0.006
Gender								
Female	163	49.7	44.1–55.2	48/130	36.9	16/97	16.5	0.001
Male	165	50.3	44.7–55.8	55/128	43.0	17/91	18.7	0.0003
Location								
Bindo	69	21.0	16.7–25.8	6/52	11.5	1/39	2.6	–
Zilé-PK	259	79.0	74.1–83.2	97/206	47.1	32/149	21.5	< 0.0001

^a^Chi-square test to compare proportion of schistosomiasis cases between end of phase 1 and end of phase 2

*Abbreviations*: n, number of schistosomiasis cases; N, number of participants; CI, confidence interval

### *Schistosoma* infection morbidity

**Fig. 2:**
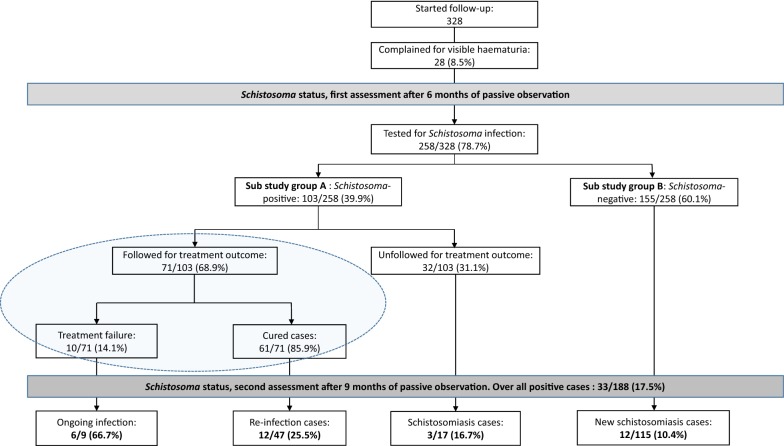
Study participants flowchart. The ellipse indicates participants monitored for praziquantel treatment outcome. For each proportion figure calculated, the denominator represents the number of subjects included (i.e. the numerator from the previous step)

As depicted in Fig. 2, among the 328 participants who entered the follow-up phase, 258 (78.7%) were tested for schistosomiasis at the end of the phase 1. During that phase and before the first assessment of schistosomiasis status, 28 (8.5%) participants complained about visible haematuria which was confirmed by Combur test (04510062171). These cases were positive for urine filtration and therefore confirmed as heavy *Schistosoma* infections, and were treated with PZQ. Hence, the haematuria incidence was 0.12 person-years in the cohort. At first assessment, a total of 103 participants (study sub-group A) were found to be infected with *Schistosoma* spp., resulting in 40% (95% CI: 34–46%) of the study population with schistosomiasis. Heavy infection intensity accounted for 45% (46/103). As presented in Table 1, schistosomiasis was more prevalent in Zilé-PK compared to Bindo (47% *vs* 11%, *χ*^2^ = 20.419, *df* = 1, *P *< 0.0001). However, there was no evidence of a difference in the percentage of schistosomiasis cases between males and females (43 *vs* 37%, *χ*^2^ = 0.747, *df* = 1, *P* = 0.39).

At the second assessment, a total of 33 participants out of the 188 present at that time point were found to be positive, yielding 17% of the study population with schistosomiasis. Heavy infection intensity accounted for 12% (4/33). Compared to the first round of treatment, no statistically significant improvement was observed among children aged 6–8 years-old (26 *vs* 23%, *χ*^2^ = 0.032, *df* = 1, *P* = 0.86), in contrast to other age groups where a statistically significant decrease in percentage of schistosomiasis cases was observed (Table 2). A similar decrease in percentage of schistosomiasis cases was observed for gender with 37 *vs* 16% (*χ*^2^ = 10.46, *df* = 1, *P* = 0.001) for females and 43 *vs* 19% (*χ*^2^ = 13.14, *df* = 1, *P* = 0.0003) for males. With regard to location, only one (3%) case of schistosomiasis among the 39 participants assessed was found at Bindo, while 21% (32/149) of schistosomiasis cases were found in Zilé-PK area, reflecting a significant decrease in percentage of schistosomiasis cases for both locations (*χ*^2^ = 23.42, *df* = 1, *P *< 0.0001) compared to the first assessment. The sub-study group B (which included participants negative at first assessment) allowed us to estimate schistosomiasis incidence in our study cohort. Among this sub-population, 12 new schistosomiasis cases out of the 115 participants evaluated at the second assessment were recorded, yielding a 10% cumulative incidence or 0.17 person-year incidence of schistosomiasis.

**Table 2:** Distribution of ERR and CR among the study population and by infection intensity

	Egg reduction rate (ERR)							Cure rate (CR)						
	Post dose 1			Post-dose 3			*P*-value^c^	Post-dose 1			Post-dose 3			*P*-value^c^
	e/E^a^	%	*P*-value	e/E^a^	%	*P*-value		*n*/*N*^b^	%	*P*-value	*n*/*N*^b^	%	*P*-value	
Study population	309/4369	92.9		408/7953	94.9	–	0.003	52/67	77.6		72/82	87.8	–	0.15
Age			< 0.0001			< 0.0001	< 0.0001			0.95^d^			0.91	0.78
6–8	5/313	98.4		7/2017	99.7			10/12	83.3		13/15	86.7		
9–11	69/1727	96.0		70/2739	97.4			19/25	76.0		24/27	92.0		
12–14	223/1420	84.3		99/1514	93.5			16/21	76.2		21/25	88.9		
15–23	12/909	98.7		232/1683	86.2			7/9	77.8		14/15	93.3		
Gender			< 0.0001			< 0.0001	< 0.0001			0.058			0.09	0.007
Female	7/1914	99.6		41/4602	99.1			27/30	90.0		39/41	95.1		
Male	302/2455	87.7		367/3351	89.0			25/37	67.6		33/41	80.5		
Location						0.020								
Bindo	0/1	100		38/512	92.6			1/1	100		3/4	75.0		
Zilé-PK	309/4368	92.9		370/7441	95.0		0.0001	51/66	77.3		69/78	88.5		0.12
Infection intensity			< 0.0001			< 0.0001	< 0.0001			0.01^d^			0.004^d^	< 0.0001
Light	57/519	89.0		2/566	99.6			40/46	87.0		45/46	97.8		
Heavy	252/3850	93.5		406/7387	94.5			12/21	57.1		27/36	75.0		

^a^E is the total *Schistosoma* egg counts at baseline and e is the total *Schistosoma* egg counts at control

^b^N is the number of participants treated at baseline and n is the number of participants found negative at control

^c^*P*-value to assess the significant difference observed between post-dose 1 and post-dose 3 results

^d^Fisher’s exact test applied

*Note*: The ERR and CR was assessed at PZQ post-dose1 (*n* = 67) and post-dose 3 (*n* = 82)

### PZQ administration

Among the 115 participants that were found to be positive at least once for schistosomiasis, 103 were detected positive at the first assessment and 12 at the end of the follow-up phase. A total of 112 (97%) were treated with PZQ. Of these, 106 (92%) and 100 (89%) completed their second and third doses of treatment, respectively. The PZQ doses administered ranged from 38 mg/kg body weight to 65 mg/kg body weight, with a mean (± SD) of 56.8 ± 6.9 mg/kg body weight. The mean time (± SD) between the first and second dose, and between the second and the third dose was 5.6 ± 1.5 and 4.6 ± 1.7 weeks, respectively. The mean time (± SD) between the first dose, the first control and between the last dose and the last control among those remaining positive was 4.7 ± 0.9 and 3.3 ± 1.3 weeks, respectively. In addition, during the treatment phase we recorded one case of vomiting in the first hour after the first-dose administration.

### Outcome of praziquantel treatment

Data for assessment of PZQ treatment outcome were available for 67 and 82 infected participants after the first and third doses of treatment, respectively. Outcomes are presented in Table 2. We found ERR of 93% and 95% after the first and the third dose of PZQ, respectively. The ERR was significantly lower for males compared with females after the first (88 *vs* 100%, *χ*^2^ = 231.31, *df* = 1, *P *< 0.0001) and the third (89 *vs* 99%, *χ*^2^ = 401.23, *df* = 1, *P *< 0.0001) PZQ administration. With regard to the intensity of the disease, the ERR was significantly lower for light than heavy infection intensity after the first PZQ administration (89 *vs* 93%, *χ*^2^ = 13.701, *df* = 1, *P* = 0.0002) but was significantly higher after the third PZQ administration (100 *vs* 94%, *χ*^2^ = 28.569, *df* = 1, *P *< 0.0001).

**Fig. 3:**
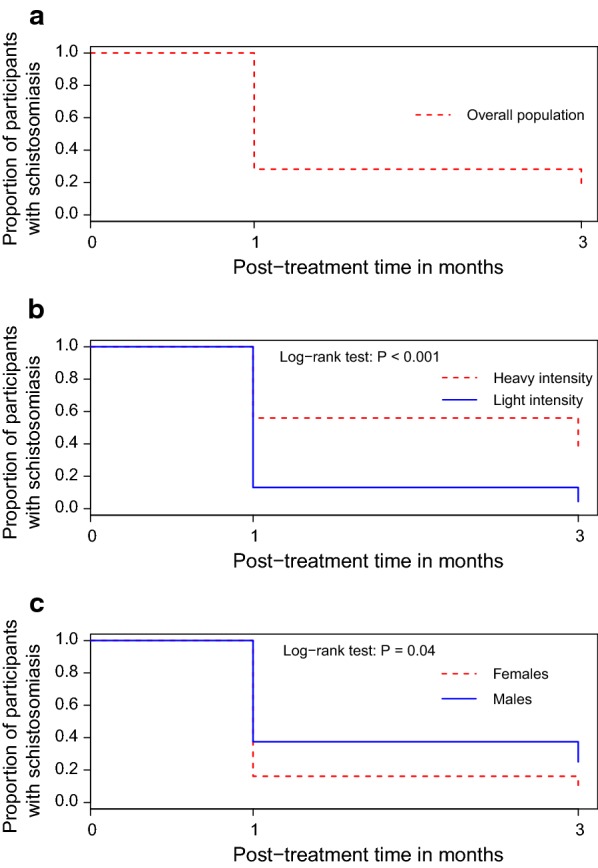
Kaplan Meier curves showing the probability to cure one month after the first and the third dose of praziquantel, respectively, among the general study population (**a**), per infection intensity (**b**) and per gender (**c**)

In addition to the EER, we found an overall CR of 78% and 88% after the first and the third dose of PZQ, respectively. The CR was somewhat lower for males compared with females after the first (68 *vs* 90%, *χ*^2^ = 3.594, *df* = 1, *P* = 0.058) and third (80 *vs* 95%, *χ*^2^ = 2.847, *df* = 1, *P* = 0.09) PZQ administration, respectively, but no statistically significant difference was detected. In contrast to ERR for infection intensity, we found a higher CR among participants with light infection intensity compared to their counterparts with heavy infection intensity after the first (87 *vs* 57%, Fisher’s exact test: *P* = 0.01) and the third (98 *vs* 75%, Fisher’s exact test: *P* = 0.004) PZQ administration, respectively. As depicted in Fig. 3, the probability of cure is significantly higher in female than in male patients (Log-rank test: *P* = 0.04), and for individuals with light infection intensity than for those with heavy infection intensity (Log-rank test: *P *< 0.001) during the whole treatment phase.

### Schistosomiasis reinfection

Among the participants who received the full PZQ regimen, post-treatment infection status was assessed for a total of 82 subjects, including 71 during the first follow-up phase and 11 at the end of the second follow-up phase. As depicted in Fig. 2, among the 71 participants followed for treatment outcome after the first treatment round, 10 (14%) remained positive for eggs in urine one month after the last dose of PZQ. Out of these 10 participants, three became negative at the end of the second follow-up phase. Of the other 61 (86%) participants who became negative (no eggs detected in their urine samples) after the full drug regimen at the first assessment, 12 out of the 47 that were followed-up to the end of the second follow-up period developed schistosomiasis, yielding a reinfection rate of 25%. The mean time to *Schistosoma* reinfection of these 12 participants was 44.6 weeks.

## Discussion

A main objective of this study was to describe the current morbidity of schistosomiasis in our study population. We therefore looked for prevalence, incidence and intensity of the disease as indicators of morbidity, using different diagnostic tools, namely eggs in fresh urine or self-reported visible haematuria. In terms of prevalence, the percentage of schistosomiasis cases we found based on urine filtration reflects the prevalence usually reported from the area. Indeed, although at the time of schistosomiasis assessment some participants who were initially included in the study cohort had withdrawn by the end of the follow-up period, 47% and 11% of schistosomiasis cases that were found in our study cohort for Zilé-PK villages and Bindo, respectively, are comparable to 41% or 43% prevalence for Zilé-PK and 15% or 19% prevalence for Bindo previously found in 2012 [22] and 2014 [23], respectively. These results show that prevalence remains stable and moderate over time in these communities. In addition to the prevalence, to the best of our knowledge the present study also assessed schistosomiasis incidence in the study area for the first time. Eight percent of the study population with visible haematuria were all confirmed as schistosomiasis cases, yielding a 0.12 person-year incidence of self-reported visible haematuria, when taking into account each participant follow-up time across all follow-up periods. Based on the urine filtration technique, 10% of the participants who were egg-negative during the first schistosomiasis assessment were found to be egg-positive during the second schistosomiasis assessment, resulting in a 0.17 per person-year schistosomiasis incidence. To our knowledge, the present study also describes an estimation of infection intensity for the first time. When considering only the first cases of schistosomiasis per participant, about half (46%) of the *Schistosoma* infections were heavy. Heavy schistosomiasis infection is indicative of a high parasite load and is associated with frequent or long-standing *Schistosoma* exposure [27, 28]. One out of two participants with schistosomiasis, and more males than females, can be assumed to be constantly exposed to a transmission hotspot, most likely due to their daily activities such washing, bathing, swimming or fishing.

The second-most important objective of this study was to report the outcome of schistosomiasis treatment with PZQ. Treatment was intended to be administered with 60 mg/kg body weight. Using 600 mg scored tablets, accurate dosage according to the participants’ weight was difficult to reach. In addition, some participants accidentally received a dosage different to what had been calculated. Taking this into account, a mean dosage of 57 mg/kg body weight was given, with moderate variation (SD = 7). The drugs were well tolerated; however, as reported above, one participant, a 14-year-old girl weighting 44 kg, vomited less than one hour after having received 4.5 tablets of 600 mg of PZQ. PZQ is indeed commonly reported to be safe [5, 29]. The results of this study show that PZQ efficacy was satisfactory even after the first dose of treatment, as indicated by an ERR of more than 90%. This result is in line with a satisfactory PZQ efficacy reported in several countries in Africa for treatment of *S. haematobium* infection [30–32] as well as for *S. mansoni* [29, 30, 33], although in these studies the regimen was 40 mg/kg. However, in contrast, other studies have reported a doubtful efficacy of PZQ in school children [3]. Factors, such as gender [3], prevalence [4] and intensity of the infection [31] have been found to affect the efficacy of PZQ. In the present study, PZQ efficacy was lower in male than female patients, and for heavy than light infection intensities. This result corroborates the finding of Kabuyaya et al. [3], who in 2017 reported a higher ERR in females compared to male school children aged from 10–15 years-old living in South Africa, even after two doses of PZQ. Interestingly, a higher ERR was found in participants with heavy infections compared to those with light infections. This finding could be explained by the capacity of PZQ to consistently reduce egg excretion through elimination of adult worms, as sustained by the overall ERR we found. However, in both groups a number of participants continued to excrete *Schistosoma* eggs, particularly in those with high-infection intensity, even after three doses of PZQ. Instead of a possible PZQ resistance, we hypothesize that these participants still excrete eggs after treatment, probably because of the schistosomulae present at the time of treatment, or because of the very early reinfection, both scenarios are consecutive to frequent parasite exposure.

Schistosomiasis reinfection is common in areas with moderate or high risk [3, 4]. In the present study, a 25% reinfection rate at 9 months post-treatment was observed. This is higher than what has been reported by Senghor et al. [34] in 2015 among children living in a low-transmission area in Senegal two to three months after treatment, and less than what was found at 12 months post-treatment in 1992 by Ofoezie et al. [32] among children living in Nigeria. Although the reinfection pattern varies with location as demonstrated by N’goran et al. [4] among schoolchildren in three neighboring villages in the Ivory Coast, the reinfection rate was reported to increase over time. Indeed, in a study conducted among children in Nigeria, authors reported an increase of reinfection rates over time from 9% at three months post-treatment to 39% at one year post-treatment, respectively [32]. Our results suggest that reinfection occurs early in the study population. This assumption is supported by the fact that 15% of our participants treated for schistosomiasis remained positive for the presence of eggs in urine even during the three month-treatment phase, and six out of nine of them remained positive up to about one-year post-administration from the first dose of PZQ. Although the hypothesis of PZQ resistance is possible, we assume these cases are frequent reinfection cases; and hypothesize that some people in our study area are continuously exposed to *Schistosoma* spp. due to the proximity of their homes to freshwater bodies, and their daily activities. In that case, the risk of reinfection will be continuous. Our results argue in favour of unequal exposure of the population to schistosomiasis. A higher proportion of schistosomiasis cases found among males indicates their increased exposure to the parasite than females. Indeed, more engagement in water-contact activities of males was suggested by Onifade et al. [35] to explain the same effect observed among school aged children living in an endemic area of Nigeria. In any case, at the end of study follow-up and as presented in Table 3, three kinds of population groups stand out, which can be discriminated according to the potential level of exposure to schistosomiasis: (i) those who are not exposed to schistosomiasis, meaning that they are not in contact with freshwater bodies, and who can be identified in our study as those who remain negative during the whole survey; (ii) those who are accidentally or occasionally in contact with schistosomiasis foci and can be identified in our survey as those who remained negative during the follow-up after treatment; and (iii) those who are frequently exposed to the parasite, probably due to their daily activities such as bathing or household work known to be associated with a high risk of infection [36]. This last group could be identified in our study population as those who remained positive despite administration of PZQ multiple times, and those found re-infected early after being considered as cured. Therefore, the application of PZQ treatment should be different with regard to the level of exposure. Indeed, if there is no role for untargeted PZQ treatment for the first group cited below, the objective of the treatment for the second group would be to achieve a cure status. In the third group, if the cure is not the main objective due to the high risk of reinfection, repetitive treatment at least once a year during their lifetime exposure will reduce at least the morbidity of the disease, and will be beneficial throughout their adulthood, as reported by WHO [5, 7]. In this scenario, we therefore recommend to complement large scale treatment with education about frequent freshwater contact so that individuals with frequent freshwater contact should then be able to identify themselves and ask for free treatment at least once a year until they leave the endemic area. As mentioned above, artemether is nowadays suggested to be of use to prevent schistosomiasis infection or reinfection [11, 13]; however, it cannot be recommended in our study area where malaria is endemic.

**Table 3:** Suggestions for recommendation of praziquantel treatment according to the potential exposure level of the population to *Schistosoma* spp.

Population group	Potential level of exposure to *Schistosoma* spp.	Suggestion of the application of praziquantel treatment	Control strategy objective
(i)	No contact with freshwater bodies	No intervention required	–
(ii)	Accidental or occasional contact with freshwater bodies	Provide praziquantel in case of haematuria and one last dose when leaving the area	To cure and prevent morbidity
(iii)	Daily or frequent contact with freshwater bodies	Educate people to seek treatment once a year during their stay in the endemic area and one last dose when leaving the area	To prevent morbidity. Only the last dose will intend to cure

Three participants treated for schistosomiasis who remained positive one month after the third dose of PZQ were found to be negative during the second assessment without any other intervention, raising the issue of *Schistosoma* eggs release following an efficient (adult worm-killing) treatment. Indeed, it has been reported that eggs can still be released up to six weeks following PZQ treatment [37]. Therefore, with the outcome of PZQ efficacy assessed four weeks post-treatment as it was done in the present study, the results could possibly be affected by false-positive cases. This differential misclassification bias could result in an underestimation of both ERR and CR. Assessing the viability of eggs released after treatment should allow for controlling this potential bias, but was not done in the present study. However, with regard to the ERR, this should not affect the conclusion drawn on the efficacy of PZQ since the 90% threshold set by WHO to conclude for PZQ satisfactory efficacy [19] was reached. On the other hand, we report a variation of PZQ dosage administered to our study population. We have therefore assessed the outcome of PZQ treatment in an intention-to-treat approach so that instead of efficacy, we report here on the effectiveness of PZQ. Furthermore, the longitudinal study design of the present study enabled us to evoke the dynamics of schistosomiasis in the study cohort, notably the incidence of the infection, which is rarely assessed due to the fact that the accurate starting time point of an infection is difficult to determine.

## Conclusions

The present study confirms a moderate urogenital schistosomiasis prevalence in our community, where part of the population bears the main burden of the disease. Our results highlight different infection patterns which need to be identified and described in order to enable appropriate schistosomiasis control. In a community where snail habitats and human freshwater contact are difficult to control and where PZQ effectiveness is reported, morbidity control should remain a priority particularly for a population with a high risk of exposure. Administration of PZQ in this sub-population should be tailored. Instead of MDA, self-administration of PZQ once a year for people at high risk might be a viable alternative.

## Supplementary information

**Additional file 1: Table S1.** Name and modalities of each variable included in the analysis. **Table S2.** Database used for the longitudinal analysis. **Table S3.** Database used for drug efficacy assessment. **Table S4.** Database used for the survival analysis.

## Data Availability

Data supporting the conclusions of this article are included within the article. The datasets generated and analyzed during the present study are included in Additional file 1.

## Abbreviations

CEI: Comité d’Ethique Institutionnel
CERMEL: Centre de Recherches Médicales de Lambaréné
CR: cure rate
ERR: eggs reduction rate
MDA: mass drug administration
PZQ: praziquantel
WASH: water, sanitation and hygiene
WHO: World Health Organization
