# Correction to: *Schistosoma haematobium* infection morbidity, praziquantel effectiveness and reinfection rate among children and young adults in Gabon

**DOI:** 10.1186/s13071-021-04815-1

**Published:** 2021-06-21

**Authors:** Jean Claude Dejon-Agobé, Jean Ronald Edoa, Yabo Josiane Honkpehedji, Jeannot Fréjus Zinsou, Bayodé Roméo Adégbitè, Mirabeau Mbong Ngwese, Ance Mangaboula, Bertrand Lell, Tamirat Gebru Woldearegai, Martin Peter Grobusch, Benjamin Mordmüller, Ayôla Akim Adegnika

**Affiliations:** 1 Centre de Recherches Médicales de Lambaréné, Lambaréné, Gabon; 2 Center of Tropical Medicine and Travel Medicine, Department of Infectious Diseases, Division of Internal Medicine, Amsterdam University Medical Centers, Location AMC, University of Amsterdam, Amsterdam, The Netherlands; 3 Department of Parasitology, Leiden University Medical Center, Leiden, The Netherlands; 4 Division of Infectious Diseases and Tropical Medicine, Department of Medicine 1, Medical University of Vienna, Vienna, Austria; 5 Institut für Tropenmedi- zin, Eberhard Karls Universität Tübingen, Partner Site, Tübingen, Germany; 6 German Center for Infection Research, Tübingen, Germany

## Correction to: Parasites Vectors (2019) 12:577 10.1186/s13071-019-3836-6

Following publication of the original article [1], it was brought to our attention that Tamirat Gebru Woldearegai had not been added to the author list. The data published in this manuscript came from work carried out within the framework of a consortium of which Tamirat Gebru Woldearegai was a member, and so TGW should be included in the author list.

The author list has been corrected in the original article and the updated author list may be found in this correction. In addition, in relation to this correction, the Author Contributions statement has been updated as follows:

“JCDA performed clinical investigation, formal analysis, and wrote the original draft. JRE, YJH, JFZ and BRA performed clinical investigations. MN wrote the original draft of the laboratory methods. AM performed the study area map (Fig. 1). BL validated the statistical analysis. MPG supervised the study conduct. TGW, BM and AAA conceptualized the study design, and supervised the study conduct. All authors contributed to the writing of the manuscript. All authors read and approved the final manuscript.”

The authors apologize for any inconvenience caused.
